# Longitudinal accelerated brain age in mild cognitive impairment and Alzheimer’s disease

**DOI:** 10.3389/fnagi.2024.1433426

**Published:** 2024-10-22

**Authors:** Maria Ly, Gary Yu, Sang Joon Son, Tharick Pascoal, Helmet T. Karim, the Alzheimer’s disease Neuroimaging Initiative

**Affiliations:** ^1^Department of Internal Medicine, Allegheny General Hospital, Pittsburgh, PA, United States; ^2^Department of Psychiatry, Ajou University School of Medicine, Suwon, Republic of Korea; ^3^Department of Psychiatry, University of Pittsburgh, Pittsburgh, PA, United States; ^4^Department of Neurology, University of Pittsburgh, Pittsburgh, PA, United States; ^5^Department of Bioengineering, University of Pittsburgh, Pittsburgh, PA, United States

**Keywords:** Alzheimer’s disease, ADNI, brain age, trajectories, MCI

## Abstract

**Introduction:**

Brain age is a machine learning-derived estimate that captures lower brain volume. Previous studies have found that brain age is significantly higher in mild cognitive impairment and Alzheimer’s disease (AD) compared to healthy controls. Few studies have investigated changes in brain age longitudinally in MCI and AD. We hypothesized that individuals with MCI and AD would show heightened brain age over time and across the lifespan. We also hypothesized that both MCI and AD would show faster rates of brain aging (higher slopes) over time compared to healthy controls.

**Methods:**

We utilized data from an archival dataset, mainly Alzheimer’s disease Neuroimaging Initiative (ADNI) 1 with 3Tesla (3 T) data which totaled 677 scans from 183 participants. This constitutes a secondary data analysis on existing data. We included control participants (healthy controls or HC), individuals with MCI, and individuals with AD. We predicted brain age using a pre-trained model and tested for accuracy. We investigated cross-sectional differences in brain age by group [healthy controls or HC, mild cognitive impairment (MCI), and AD]. We conducted longitudinal modeling of age and brain age by group using time from baseline in one model and chronological age in another model.

**Results:**

We predicted brain age with a mean absolute error (MAE) < 5 years. Brain age was associated with age across the study and individuals with MCI and AD had greater brain age on average. We found that the MCI group had significantly higher rates of change in brain age over time compared to the HC group regardless of individual chronologic age, while the AD group did not differ in rate of brain age change.

**Discussion:**

We replicated past studies that showed that MCI and AD had greater brain age than HC. We additionally found that this was true over time, both groups showed higher brain age longitudinally. Contrary to our hypothesis, we found that the MCI, but not the AD group, showed faster rates of brain aging. We essentially found that while the MCI group was actively experiencing faster rates of brain aging, the AD group may have already experienced this acceleration (as they show higher brain age). Individuals with MCI may experience higher rates of brain aging than AD and controls. AD may represent a homeostatic endpoint after significant neurodegeneration. Future work may focus on individuals with MCI as one potential therapeutic option is to alter rates of brain aging, which ultimately may slow cognitive decline in the long-term.

## 1 Introduction

Identifying individuals at risk for developing Alzheimer’s disease (AD) may improve therapeutic interventions and prevention efforts before irreversible neurodegenerative changes occur. Prior clinical trials have partially failed as they attempted to intervene too late (Sperling et al., 2011). Early identification of AD can be complicated by the wide variability in the normal aging process (Arenaza-Urquijo and Vemuri, 2018). Several factors contribute to this variability including early genetic and environmental exposures, cellular and tissue dysfunction, reactive oxide species, and modifiable risk factors (e.g., insulin resistance, cardiometabolic risk, inflammation, obesity). These can ultimately alter an individual’s brain structural reserve (Stern et al., 2019), putting them at higher risk for AD. Recently, studies have shown that brain age—a measure of the age of an individual’s brain using machine learning—is correlated with cognitive impairment, AD, traumatic brain injury, and mortality amongst many other disorders (Ly et al., 2020; Cole and Franke, 2017; Cole et al., 2015; Cole et al., 2018; Liem et al., 2017).

We previously developed a machine learning algorithm to estimate brain age based on gray matter volume in healthy individuals without significant brain amyloid (Ly et al., 2020). This algorithm predicts an individual’s age from their gray matter volume—if an individual has a predicted age greater than their chronologic age, this implies that they may have some differences compared healthy adults their chronological age. Our model showed higher accuracy compared to other amyloid-insensitive models in predicting age (Ly et al., 2020). Unlike models that were not trained on amyloid-negative individuals, our model was able to differentiate between AD diagnostic groups (amyloid-negative cognitively normal, amyloid-positive cognitively normal, early mild cognitive impairment, late mild cognitive impairment, and AD) in a cross-sectional cohort (Ly et al., 2020). We recently replicated our results in a separate South Korean cohort (*n* = 650), and we further demonstrated that baseline brain age was predictive of future cognitive decline (Karim et al., 2022). These results show that brain age is not only a useful correlate of gray matter volume but may also help identify those at high risk of atrophy and cognitive decline over time.

One major limitation of the current literature is the scarcity of longitudinal studies. Most past research has concentrated on cross-sectional comparisons finding that brain age is greater in MCI and AD compared to cognitively healthy individuals (Ly et al., 2020; Liem et al., 2017; Karim et al., 2022; Beheshti et al., 2018; Gaser et al., 2013). However, there are relatively few studies investigating how brain age changes over time in these groups. One study found that participants who converted to AD within 3 years had greater brain age by 3 years on follow-up compared to baseline (Franke and Gaser, 2012). Another study showed that participants who progressed to AD from MCI had a faster brain aging trajectory compared to cognitively normal individuals or individuals with stable MCI (Taylor et al., 2022). In one study, participants with AD had accelerated neurodegeneration in the hippocampus, amygdala, middle temporal gyrus, entorhinal cortex, and several other regions beginning as early as the 4th decade of life (Planche et al., 2022). In this analysis, we evaluated longitudinal trajectories of brain age in ADNI 1 using our amyloid-sensitive model.

In this study, we have applied our previously validated brain age prediction model (Ly et al., 2020; Karim et al., 2022). We hypothesized that the MCI and AD groups would show higher brain age compared to HC. We also hypothesized that the MCI and AD groups would have faster rates of brain age over time. We fit longitudinal mixed effect models with brain age as the outcome and age, sex, education, race, and clinical group as independent predictors or covariates. We conducted analyses with age as a time-dependent effect as well as time from baseline scan separately. These analyses address: (Sperling et al., 2011) do the groups differ in rates of brain age change across older adults (model 2) and (Arenaza-Urquijo and Vemuri, 2018) do the groups differ in rates of brain age relative to their first MR scan regardless of individual chronologic age (model 3), respectively. The major difference is that model 3 attempts to investigate whether there is accelerated aging regardless of their baseline age at study entry while model 2 attempts to investigate if there is accelerated aging across different ages (e.g., at 65 vs. at 80, etc.). The current analysis replicates the results of Franke and Gaser (2012) in model 3, but extends the work by looking at chronological age trajectories in model 2.

## 2 Methods

### 2.1 Participants

This study included a total of 183 participants with 3.0 T MRI scans from the Alzheimer’s disease Neuroimaging Initiative-1 (ADNI-1).^1^ As such, the investigators within the ADNI contributed to the design and implementation of ADNI and/or provided data but did not participate in analysis or writing of this report. A complete listing of ADNI investigators can be found on the ADNI website. All participants gave written informed consent prior to participation and these studies were approved by the appropriate institutional review boards (IRB) at multiple institutions. We utilized this publicly available data as part of our analysis. We included only 3 T scans as this can affect estimation of brain age algorithms as Franke et al. has reported (Franke and Gaser, 2012). In total, this analysis included 677 scans with participants contributing a median of 4 scans.

### 2.2 MRI acquisition

Data from ADNI has been previously described (Jack et al., 2008), but we also describe the data briefly. In general, participants received an MR scan lasting anywhere from 30 min to 1 h. Various sites had different coils and MR sequence parameters, but most collected an MPRAGE with repetition time (TR) = 2,300 ms to 3,000 ms, flip angle (FA) = 8 to 9 deg., inversion time (TI) = 853–900 ms, and 160–170 slices with a roughly 1.2 mm isotropic voxel on a 3 T scanner.

### 2.3 Image preprocessing and brain age prediction model

The following has been detailed in prior work (Ly et al., 2020; Karim et al., 2022). Overall, we first segmented every image, generated a study-specific template, coregistered all images to that study-specific template, and computed the gray matter volume image for each participant. This volume image was input into the pre-trained machine learning model. This uses data from three studies to predict the age associated with each scan after conducting standard intensity scaling and computing a similarity kernel. This is described in greater detail below.

Statistical Parametric Mapping (SPM12) software was used to segment structural images into six tissue classes: gray, white, CSF, skull, soft tissue, and air (Ashburner and Friston, 2005). We then utilized the non-linear fast diffeomorphic registration DARTEL algorithm to generate a study-specific template (Ashburner, 2007). This process coregistered images to an average template and generated consecutively smoother templates that they were iteratively coregistered to. This average template was generated with the current dataset, which is what we have done previously. For this process, we used the baseline scan of all participants to first generate a study specific template using DARTEL. We visually inspected this template to ensure no issues with template generation. We then explored whether this template differed from the template that was used in the training data. To do so, we computed the correlation between voxels in the study template for these participants (*n* = 183) and the one used in the training sample (*n* = 757). We found that they were correlated with the template in the training set (Pearson *r* = 0.89, correlation between voxels in study specific template and the template of the training sample). Each image for each scan was then normalized to this generated template space and transformed into a gray matter image that preserved the total amount of gray matter by multiplying by the determinant of the Jacobian of the transformations. Images were normalized to MNI (Montreal Neurological Institute) space at 1 mm (Stern et al., 2019) isotropic resolution and then smoothed with a full-width at half-maximum of 4 mm. These images were input into our brain age algorithm.

These smoothed gray matter volume images were then input into a pre-trained machine learning algorithm (Ly et al., 2020). We have previously validated a machine learning algorithm (Ly et al., 2020; Karim et al., 2022) that predicts chronological age with gray matter volume images using the Pattern Recognition for Neuroimaging Toolbox (Schrouff et al., 2013). Mean-centered gray matter volumes were used to calculate similarity matrix kernel using the dot product (LaConte et al., 2005). The training set utilized in the original machine learning algorithm consisted of 757 individuals from publicly available databases (IXI *n* = 264, OASIS-3 *n* = 401, ADNI-3 *n* = 92), which did not have any overlap with the individuals in this present study. Description of these studies as well as demographic information is in the supplement and Supplementary Table 1. We provide a more detailed description of the training of this initial model in the original manuscript (Ly et al., 2020) as well as the Supplementary methods. Individuals had no psychiatric or neurologic disorders and no Alzheimer’s pathology as measured by positron emission tomography. The cohort was used as a covariate of no interest. The present data was not used in the training of the model, only this past pre-trained model was used to predict chronological age of this sample. This approach uses gray matter volumes and not white matter, so it could more accurately be described as a “gray matter” age marker. We use the term ‘brain age’ to refer to the measure throughout the manuscript. We additionally adjusted for the intercept and slope (i.e., subtract intercept and divide by slope) of the original brain age model as this has been identified as potentially resulting in bias of brain age estimates.

### 2.4 Statistical analysis

All analyses were performed in JMP Pro 16 (SAS Institute Inc., Cary, NC, USA). We generally conducted three models: Model 1 was for cross-sectional data, model 2 was for longitudinal data using age as a repeated measures variable, and model 3 was for longitudinal data using time from baseline scan as a repeated measures variable. We corrected the omnibus models to adjust for multiple comparisons using a Bonferroni correction of 0.05/3 = 0.0167.

#### 2.4.1 Model 1

Cross-sectionally, a multiple linear regression model was run featuring baseline brain age (first scan chronologically) for each individual as the outcome, with baseline chronological age (years), sex (female reference), education (years), race (White reference), and clinical group (HC, MCI, and AD) as independent predictors. Interaction effects between age and clinical group were also modeled.

#### 2.4.2 Model 2

Longitudinally, mixed effect models were run with brain age as the outcome and age, sex, education, race, and clinical group as independent predictors. To account for repeated individual scans, random slope and intercept effects of individual participants were modeled as well using age as a time-dependent variable. Multiple models were fit to test interactions between age and group, as well as quadratic effects of age. The Akaike Information Criterion was used to select for the best model which was ultimately reported.

#### 2.4.3 Model 3

We then conducted a similar longitudinal model, but instead used time from baseline (where 0 represented the time at baseline scan) as the predictor and the outcome was brain age minus baseline brain age. We included time, time (Arenaza-Urquijo and Vemuri, 2018), sex, education, race, group, chronologic age, and time x group interactions in the model. This approach attempts to expand upon a model in past literature (Franke and Gaser, 2012).

Model 2 and 3 used a mixed effects model with random intercepts and random slopes. Note that we evaluated higher order terms as aging may not be entirely linear especially in older adults with pathology.

## 3 Results

The demographics of our participant cohort are reported in Table 1. Of note, there was a strong predominance of men in the MCI group compared to HC and AD. Individuals in the AD group, compared to the other two groups, also had lower education on average and had fewer successive scans. The MCI and AD groups also had greater change in brain age over change in age on average. We adjust for all these differences in all analyses by including them as covariates.

**Table 1:** Demographic information for study cohort: Mean and standard deviations are reported for groups stratified by clinical groups of healthy control (HC), mild cognitive impairment (MCI), and Alzheimer’s disease (AD).

	HC	MCI	AD		*p*
*N* (183 total)	59	82	42		
Baseline age (years)	75.7 (4.9)	74.9 (8.1)	75.0 (8.6)	*F*(2,180) = 0.227	0.797
Sex	39.0% M	62.2% M	38.1% M	*χ*^2^ = 10.17	0.006
Education (years)	16.2 (2.6)	15.7 (3.2)	14.7 (3.2)	*F*(2,180) = 3.229	0.042
Race (AI/AN, Asian, Black, White)	0/1/3/55	1/1/2/78	0/0/2/40	*χ*^2^ = 3.503	0.743
Race (percentages)	0/1.7/5.1/93.2%	1.2/1.2/2.4/95.1%	0/0/4.8/95.2%		
Baseline brain age (years)	76.9 (5.1)	78.4 (4.5)	78.9 (6.0)	*F*(2,180) = 2.514	0.084
Number of scans per individual	3.8 (1.1)	4.0 (1.4)	2.9 (1.0)	*F*(2,180) = 13.332	<0.0001
Δ age (years)	2.9 (0.7)	2.0 (0.8)	1.4 (0.6)	*F*(2,168) = 16.065	<0.0001
Δ brain age/Δ age	0.9 (0.6)	1.5 (0.9)	1.6 (3.0)	*F*(2,168) = 3.68	0.027

Variables were compared between groups using one-way ANOVAs or Chi tests when appropriate with *p*-values reported. *Δ* brain age and Δ age represent differences between the first and the final scans available. AI/AN, American Indian/Alaska Native. *p*-values indicate significance.

### 3.1 Brain age model performance

Cross-sectionally at baseline, the correlation between chronological age and brain age was *r*(181) = 0.61 and *R*^2^ = 0.38 while brain age and chronological age had a mean absolute error of 3.12 years. This shows the fit of the overall brain age model. For all analyzed scans, the correlation between brain age and chronological age had a mean absolute error (MAE) of 3.15 years with *r*(675) = 0.574 and *R*^2^ = 0.329, indicating our model predicted chronological age for this sample within expected tolerance (typically MAE of <5 years).

#### 3.1.1 Model 1

**Figure 1:**
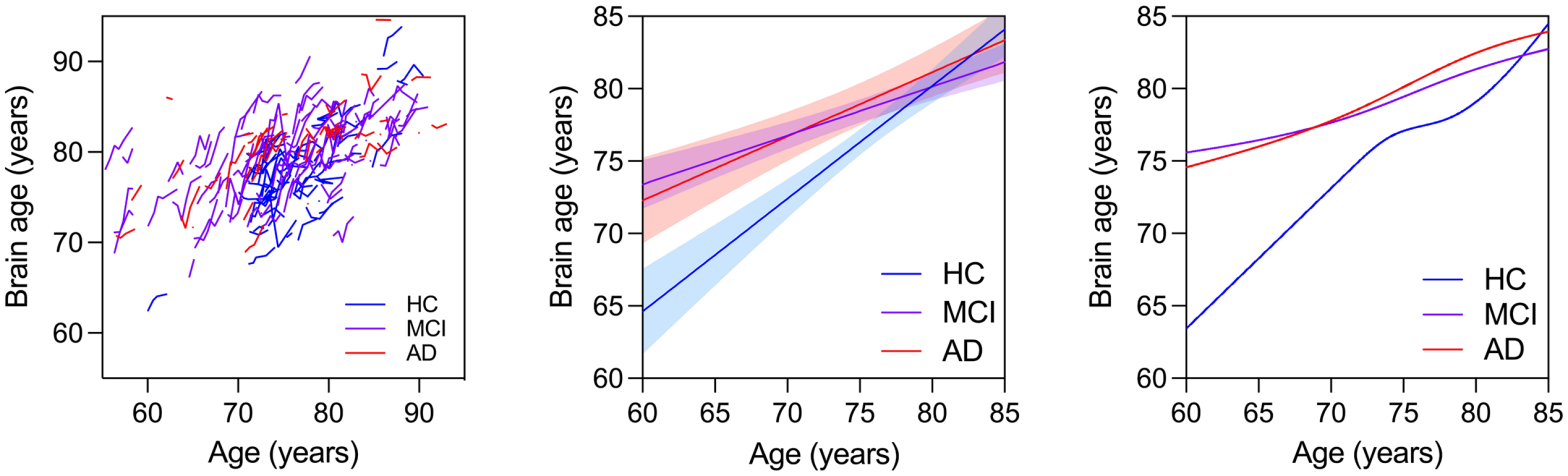
(Left) Spaghetti plot for longitudinal brain ages over age for all individuals. Trend lines for brain age against age: Linear regression lines with 95% confidence intervals are plotted over age for brain ages calculated from baseline scans for each individual cross sectionally (Middle). Cubic splines with 4 knots are plotted from longitudinal brain age series for all individuals (Right).

We first modeled the data cross-sectionally with brain age as the outcome and age, sex, education, race, group, and group x age as predictors. We found that 47.7% of the variance in brain age was explained by this model [*F*(8,174) = 19.85, *p* < 0.0001, *R*^2^ = 0.477]. Each year of age was associated with 0.506 years greater brain age (~6.1 months), while having clinical AD was associated with 0.158 years (2 months) greater brain age compared to HC. MCI by age interaction was significant, indicating younger individuals with MCI had higher brain ages than HC. These results are shown in Table 2 and Figure 1 (middle).

**Table 2:** Multiple regression model for cross sectional analysis with brain age as outcome variable for baseline (first) scans of all individuals.

Term	*β*	B (SE)	Lower 95%	Upper 95%	*t*	*p*
Intercept		37.400 (3.604)	30.287	44.513	10.38	<0.0001
**Age**	**0.727**	**0.506 (0.045)**	**0.418**	**0.595**	**11.3**	**<0.0001**
Sex (Female reference)	0.024	0.122 (0.304)	−0.478	0.722	0.4	0.6892
Education	0.056	0.094 (0.098)	−0.099	0.287	0.96	0.338
Race (White reference)	−0.108	−1.211 (0.631)	−2.455	0.034	−1.92	0.0566
MCI (HC reference)	0.078	0.461 (0.390)	−0.310	1.230	1.18	0.2395
**AD (HC reference)**	**0.158**	**1.094 (0.454)**	**0.198**	**1.990**	**2.41**	**0.017**
MCI*Age	**−0.207**	**−0.174 (0.054)**	**−0.280**	**−0.068**	**−3.24**	**0.0014**
AD*Age	−0.081	−0.084 (0.060)	−0.202	0.035	−1.39	0.1661

*F*(8,174) = 19.85, *p* < 0.0001, *R*^2^ = 0.477. B indicates unstandardized coefficients with standard errors indicated. Bolded lines indicate significant effects.

#### 3.1.2 Model 2

In this model we investigated brain age associations with age, age (Arenaza-Urquijo and Vemuri, 2018), sex, education, race, and group. We found that the best fitting models did not have the interaction terms as this showed the lowest AIC (Table 3). We thus report the effects of that model in Table 4. The linear and quadratic effects of age had significant positive and negative effects on brain age, respectively. This means that brain age increased quadratically up to a point and flattened out.

**Table 3:** Comparison of Akaike Information Criterion (AIC) scores for mixed effect models with indicated effects.

Model effects	AIC
**age, age** ^**2**^ **, group**	**2701.4736**
age, age^2^, group, age*group	2703.3403
age, age^2^, group, age^2^*group	2703.079
age, age^2^, group, age*group, age^2^*group	2703.5396
age, group	2710.5263
age, age^2^	2717.1751

All models include covariates of sex, education, and race. The main model reported in further detail is indicated with bold. Bolded lines indicate significant effects.

**Table 4:** Mixed effects model results with parameter estimates for fixed effects shown.

Term	Estimate	Std Error	95% Lower	95% Upper	*t*	*p*
Intercept	−42.01382	19.634	−80.8202	−3.207434	−2.14	0.0340
**Age**	**2.5504801**	**0.5115729**	**1.5395798**	**3.5613803**	**4.99**	**<0.0001**
**Age** ^**2**^	**−0.012717**	**0.0033535**	**−0.019343**	**−0.006091**	**−3.79**	**0.0002**
Sex (Female reference)	−0.136446	0.3324544	−0.793854	0.5209614	−0.41	0.6821
Education	0.0125676	0.1074764	−0.199978	0.2251132	0.12	0.9071
Race (White reference)	−1.149588	0.7013778	−2.537569	0.2383933	−1.64	0.1037
**MCI (HC reference)**	**0.9109336**	**0.4378985**	**0.0451438**	**1.7767235**	**2.08**	**0.0393**
**AD (HC reference)**	**1.1618634**	**0.5174461**	**0.1389412**	**2.1847855**	**2.25**	**0.0263**

Random slope and intercept effects of individual subjects were included with the model to account for longitudinally repeated nature of scans. Significant effects indicated in bold. Bolded lines indicate significant effects.

The MCI and AD groups had greater brain age compared to the HC across all ages. Cubic splines with 4 knots (Figure 1, right) were fit to longitudinal trends for individuals in each clinical group (Figure 1, left). The interaction between age on MCI or AD was not significant (not reported). These results are shown in Table 4.

#### 3.1.3 Model 3

**Figure 2:**
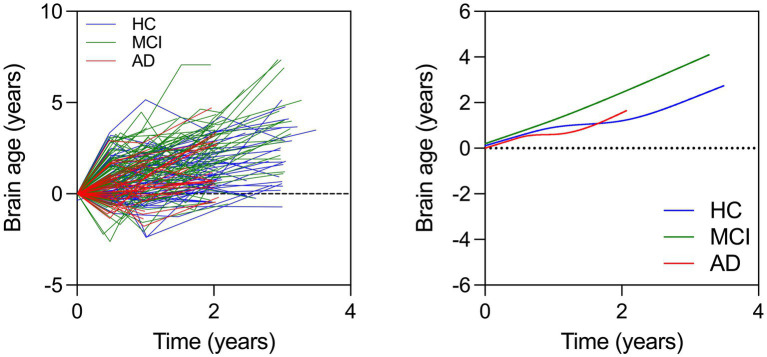
(Left) Spaghetti plot for longitudinal brain ages over time for all individuals. Cubic splines with 4 knots are plotted from longitudinal brain age series for all individuals (Right). This plot instead shows change in brain age over time in each group.

In this model, we investigated brain age associations with time (years from baseline), sex, education, race, group, and group x time interactions. We found that MCI x time interaction was significant while the AD x time interaction was not. This showed that the MCI group showed a faster rate of brain aging over time compared to the HC while the AD group did not (Table 5 and Figure 2).

**Table 5:** Mixed effects model results with parameter estimates for fixed effects shown.

Term	Estimate	Std Error	*t*	*p*
Intercept	0.5438858	0.5762681	0.94	0.3465
**time**	**1.0224886**	**0.1265497**	**8.08**	<0.0001*
time^2	−0.073252	0.0461556	−1.59	0.1132
sex	0.0204168	0.0526544	0.39	0.6987
education	−0.010467	0.0169805	−0.62	0.5385
race	0.0091528	0.1149502	0.08	0.9366
MCI	−0.022003	0.0691175	−0.32	0.7506
AD	−0.056387	0.0857215	−0.66	0.5113
age	−0.003963	0.0070717	−0.56	0.5759
time*MCI	**0.3157218**	**0.0829302**	**3.81**	0.0002*
time*AD	−0.037376	0.1131029	−0.33	0.7414

Random slope and intercept effects of individual subjects were included with the model to account for longitudinally repeated nature of scans. Significant effects indicated in bold. This model used time from baseline as a time-dependent predictor and brain age minus baseline brain age as the outcome. Bolded lines indicate significant effects.

## 4 Discussion

We applied our previously validated brain age model on an independent archival dataset evaluate changes in brain age over time. Our amyloid-sensitive model (Ly et al., 2020) predicted brain age within an acceptable margin of error. As hypothesized, we replicated past cross-sectional studies showing that AD had greater brain age than MCI which had greater brain age than HC (model 1). In our longitudinal analysis, as we hypothesized, we showed that brain ages were highest in AD, then MCI, and then HC (model 2). Contrary to our hypothesis, we found that the rate of brain age change was fastest for the MCI group compared to HC, but not for the AD group compared to HC (model 3). This is counter to our hypothesis that AD would have the greatest rate, which may indicate that brain age has a ceiling similar to atrophy—where AD represents a new setpoint with high levels of atrophy (though this remains speculative). Overall, these results indicate that the MCI group may be in a transitional phase of neurodegeneration.

Our findings partially align with those reported by Franke and Gaser (2012). They found that brain age was greatest in the AD group compared to MCI compared to HC. They also found that both the AD and MCI groups had accelerated brain aging over time. We found that the MCI group had a faster rate of brain aging over time compared to HC, but did not find that in the AD group. This suggests that MCI may represent a dynamic phase of rapid neurodegeneration. In hypothetical models of neurodegeneration, HC and AD groups might be seen as two stable homeostatic endpoints, with MCI representing the transitory phase between them. This is in line with the AD hypothetical biomarker curves (Jack et al., 2010). However, this discrepancy between our findings and that of Franke and Gaser (2012) needs further evaluation.

We found that using age as a time-dependent variable showed that the AD and MCI groups had higher brain age than HC, but did not show more rapid brain aging (i.e., model 2). However, when we examined individual brain age trajectories (i.e., model 3), the MCI group showed faster rates of brain aging compared to both HC and AD groups. Past studies that investigated patients who converted from cognitively normal to MCI or MCI to AD have identified accelerated trajectories of neurodegeneration (Franke and Gaser, 2012; Taylor et al., 2022). One study (Taylor et al., 2022) found that individuals with progressive MCI experienced faster rates of brain aging compared to stable MCI. Our results further emphasize the clinical significance of the MCI phase in the overall pathophysiology of AD.

Leung et al. (2013) found that rates of hippocampal brain atrophy was primarily driven by individuals with progressive MCI. They did not observe greater rates of hippocampal atrophy in the Alzheimer’s disease (AD) group compared to controls. These results support our model that AD may represent a homeostatic endpoint while MCI represents a phase of rapid neurodegeneration. The authors suggested that this lack of difference may also be due to low statistical power and greater heterogeneity in the AD group where some show atrophy while others do not.

There are several limitations in this study. We only used 3 T data as this can affect brain age estimations, which partially limited which participants were included in our analysis. Participant groups did differ by sex and education, which may have partially affected the results as these have been shown to be associated with brain age. For example, education has been associated with cognitive reserve, or the ability to cope with damage or pathology that may explain differences in disease progression (Wilson et al., 2019). In addition, education has been previously associated with brain age (Steffener et al., 2016). We have adjusted for sex and education across all analyses and found that including these variables did not alter statistical results. These results may not generalize to more racially diverse samples because the current dataset was primarily White. We primarily focused on analyses of individuals who at baseline were recruited into one of the three groups. Additionally, this study needs to be further validated using more data especially findings regarding AD samples. This is especially important given that the AD sample had the lowest mean follow-up, which is a major limitation in estimating individual trajectories of brain age. While we found that brain age seems to have some cap, this may actually reflect the limited number of individuals at very high ages with very high levels of atrophy. Additionally, participants had varying numbers of follow-up visits potentially making models with higher order terms for age and time (e.g., age cubed or time cubed) worse fits. Thus, more data longitudinally within participants would greatly improve our understanding of the longitudinal changes in AD and MCI.

We found that brain age was higher in participants with MCI and AD compared to HC. We found that individuals with MCI experienced more rapid changes in brain age over time compared to HC while the AD group did not. This study builds on our previous findings suggesting that MCI individuals have more rapid rates of brain aging over time. Overall, while our results do re-affirm previous paradigms of AD and brain age modeling, they also indicate a potential need to expand our focus into mid-life as a period where the pathological processes of AD are not yet fully established. This earlier stage may present a critical opportunity for interventions targeting modifiable risk factors associated with AD.

## Data Availability

Publicly available datasets were analyzed in this study. This data can be found here: https://adni.loni.usc.edu.

## Footnotes

^1^ https://adni.loni.usc.edu/
