# Supplementary material for: Longitudinal accelerated brain age in mild cognitive impairment and Alzheimer’s disease
Source: Front Aging Neurosci. 2024 Oct 22;16:1433426. doi: 10.3389/fnagi.2024.1433426 (PMC11534682; doi:10.3389/fnagi.2024.1433426)
Supplement: Supplementary file 1 [file Table_1.docx]

**Longitudinal accelerated brain age in Mild Cognitive Impairment and Alzheimer’s Disease**

**Authors.** Maria Ly, MD, PhD^1,^**^+^**, Gary Yu, MD, PhD^1,^**^+^**, Sang Joon Son, MD, PhD^2^, Tharick Pascoal, MD, PhD^3,4^, Helmet T. Karim, PhD ^3,5^*, for the Alzheimer’s disease Neuroimaging Initiative

1. Department of Internal Medicine, Allegheny General Hospital, Pittsburgh, PA, USA
2. Department of Psychiatry, Ajou University School of Medicine, Suwon, South Korea
3. Department of Psychiatry, University of Pittsburgh, Pittsburgh, PA, USA
4. Department of Neurology, University of Pittsburgh, Pittsburgh, PA, USA
5. Department of Bioengineering, University of Pittsburgh, Pittsburgh, PA, USA

**^+^** These authors contributed equally to this work

* Correspondence to:

Helmet T. Karim, PhD

Assistant Professor of Psychiatry and Bioengineering

University of Pittsburgh

email: [hek26@pitt.edu](mailto:hek26@pitt.edu)

**Keywords.** Alzheimer’s disease, ADNI, brain age, trajectories, MCI

**Supplemental Methods.**

Training Sample Description

We have previously described the training of the initial model. This model was used without any additional training on the current dataset. We provide here a description of the data included in the training set.

We used several datasets for the training set including [Alzheimer’s disease Neuroimaging Initiative (ADNI); Information eXtraction from Images (IXI); and Open Access Series of Imaging Series: Longitudinal Neuroimaging, Clinical, and Cognitive Dataset for Normal Aging and Alzheimer’s disease (OASIS-3)].

ADNI was designed for testing whether serial MRI, positron emission tomography (PET), and other biological markers were associated with cognitive differences and changes over time. The IXI dataset consists of 600 MR images from healthy adults across the lifespan. OASIS-3 is a retrospective compilation of data from approximately 1000 participants at the Washington University in Saint Louis Knight ADRC over the course of 30 years (https://www.oasis-brains.org).

All participants provided informed consent, with the approval of the Human Use Subcommittee of the Radioactive Drug Research Committees and the Institutional Review Board of the respective associated Universities.

Demographic information on the training set is in supplemental table 1.

Brain Age Model Description

For the model training, structural MPRAGE data was used to first segment images into tissue classes (gray, white, cerebrospinal fluid, skull, soft-tissue, and air) using SPM12. Similar to this study analysis, the training data was then input into DARTEL algorithm to register images to the Montreal Neurological Institute space template and then generated a template per cohort. These are then used to generate deformation fields, which are applied to each segmentation and multiplied by the Jacobian to estimate tissue volume in MNI space, these are then smoothed with a 4 mm smoothing kernel.

We used the Pattern Recognition for Neuroimaging Toolbox^18^ to develop the model. Whole brain, voxel-wise gray matter volumes in MNI space were input into this toolbox. These volumes were mean-centered and then used to compute a similarity matrix kernel using the dot product. This estimates the similarity between any 2 participants (an N x N matrix is generated). This matrix was used in a Gaussian Processes Regression with this similarity matrix as the independent data and chronological age as the dependent variable (adjusting for cohort: ADNI, IXI, or OASIS-3). This model performed accurately in two separate cohorts. In this study, we used this model to estimate brain ages in these new data without additional training.

|  | **CN-Aβ(-) training set  (N=757)** | **ADNI (N=92)** | **OASIS3 (N=401)** | **IXI (N=264)** |
| --- | --- | --- | --- | --- |
| **Chronological Age  (mean, std)** | 55.4 (17.5) | 72.5 (5.7) | 65.0 (9.4) | 34.9 (8.1) |
| **Sex (%F)** | 55.4% | 47.8% | 61.3% | 49.2% |
| **Education  (mean, std)** | 16.2 (2.5) | 17.0 (2.6) | 16.1 (2.5) | - |
| **Race  (% Not White)** | 17.4% | 13% | 16.2% | 21.1% |
| **Brain Age** | 55.5 (16.5) | 72.4 (5.7) | 65.1 (7.1) | 50.5 (5.8) |

**Supplemental Table 1.** Training set demographics: Demographic characteristics and predicted brain ages in the full training set and individual datasets of all cognitively normal, Aβ(-) individuals are shown.
